# Incorporating structure context of HA protein to improve antigenicity calculation for influenza virus A/H3N2

**DOI:** 10.1038/srep31156

**Published:** 2016-08-08

**Authors:** Jingxuan Qiu, Tianyi Qiu, Yiyan Yang, Dingfeng Wu, Zhiwei Cao

**Affiliations:** 1 Department of Bioinformatics, School of Life Sciences and Technology, Tongji University, Shanghai 200092, China

## Abstract

The rapid and consistent mutation of influenza requires frequent evaluation of antigenicity variation among newly emerged strains, during which several *in-silico* methods have been reported to facilitate the assays. In this paper, we designed a structure-based antigenicity scoring model instead of those sequence-based previously published. Protein structural context was adopted to derive the antigenicity-dominant positions, as well as the physic-chemical change of local micro-environment in correlation with antigenicity change. Then a position specific scoring matrix (PSSM) profile and local environmental change over above positions were integrated to predict the antigenicity variance. Independent testing showed a high accuracy of 0.875, and sensitivity of 0.986, with a significant ability to discover antigenic-escaping strains. When applying this model to the historical data, global and regional antigenic drift events can be successfully detected. Furthermore, two well-known vaccine failure events were clearly suggested. Therefore, this structure-context model may be particularly useful to identify those to-be-failed vaccine strains, in addition to suggest potential new vaccine strains.

As a continuous threat to public health, seasonal influenza viruses are under constant mutation to escape host immunity. Previous reports have revealed that mutations are primarily occurred on the major antigen protein hemagglutinin (HA), where antibodies to HA can provide effective protection to virus infection^1^. Thus measuring the antigenic change via hemagglutination-inhibition (HI) assay become frequent duties of World Health Organization (WHO) in recommending new vaccine strains. The titer results of HI assay can be calculated into the antigenic distance of the two strains (*D*_*ab*_)^2^, where log^−1^*D*_*ab*_ above 4 (*D*_*ab*_ > 2) is usually considered as an antigenic escape or variant^3^.

Since HI assay involves serum preparation and repetitive binding, computational prediction of antigenic variation has been highly desired in the past years. In the early 1980s, researches indicated the antigenicity change of an influenza virus was related to the amino acid mutations at certain positions of HA1 domain^4^,^5^. Soon Smith’s work characterized 44 such positions contributing to antigenic cluster transition which started the computational estimation of antigenic distance^6^. Meanwhile, Lee reported that mutational accumulation over seven amino acids among 131 positions of HA1 protein may lead to effective immune escape^7^. Liao *et al.* further reduced the antigenicity-dominant positions to 20 through a regression model based on pair-wise scoring vector^8^. Recently, an interesting paper tested different groups of “artificial sites” through linear model on a larger dataset of 203 pair-wise samples, and found that antigenic distance may relate to concurrent change in multiple regions of the HA1, and the additional positions on top of the 131 positions may still have antigenic activity^3^. The observed inconsistence was explained as that the evolutionary selective pressure may change on specific locations over time^9^.

Once the antigenicity-dominant positions are derived, counting the number of mutations seems to be indicative of the antigenic distance. But ineffective detections were often noticed when few mutations occurred on critical positions interacting with antibodies^7^. Then, incorporation of physic-chemical features was shown to significantly improve the predictive power of antigenic variation^8^,^10^,^11^,^12^. An outstanding example is PREDAC model, which calculated the physic-chemical features based on Wiley’s sites^5^ to successfully suggest the antigenic clusters of global influenza^11^. Recently, Sun’s work developed a sequence-based antigenicity scoring function via a bootstrapped ridge regression to quantify the antigenic distance between HA sequences^13^,^14^. Despite of the great success, all these sequence-based models pointed out that incorporating spatial information of HA structures, particularly those positions potentially involved into HA-antibody interactions, may further improve the predictive performance^3^,^10^,^11^. However, none of structure-based models have been reported yet.

In this study, a structure-based antigenicity scoring model was established fully considering the conformational context of HA structures. Based on the largest and most comprehensive dataset containing 3867 non-redundant HA pairs from 1968 to 2013, those potential antigenicity-dominant positions were derived considering the structural neighboring change on HA surface through iterative algorithm. Then, the micro-environmental descriptors covering physical-chemical properties of sub-epitope areas were subsequently integrated with glycosylation sites and position specific scoring matrix (PSSM) to build the antigenicity calculation model. Compared to sequence-based peers on independent testing dataset, our structure-based model shows significant ability in detecting antigenicity variants, in addition to better accuracy and sensitivity. At last our model was adopted to predict the global antigenicity drift of historical vaccine strains recommended by WHO.

## Results

### Derivation of antigenicity-dominant positions and model construction

**Figure 1:**
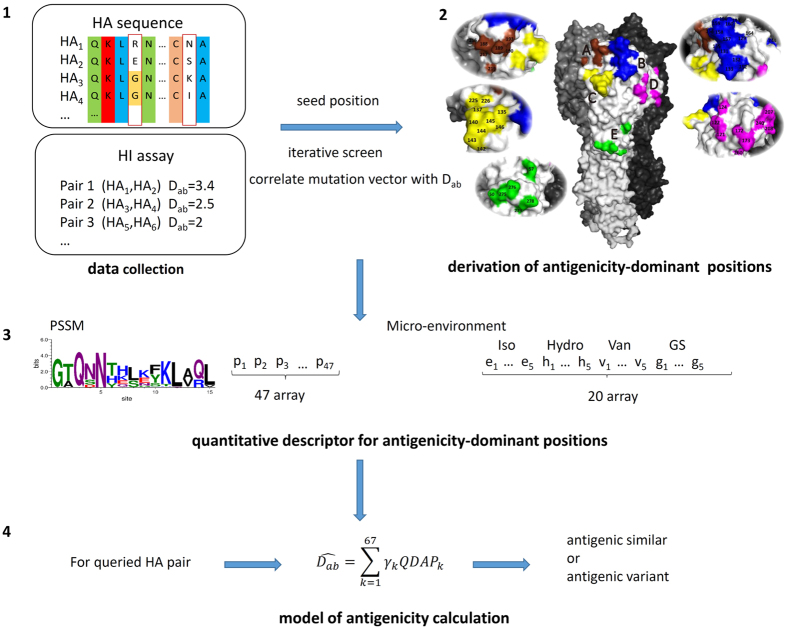
Flowchart of model. (**1**) Data collection of HI assay results and the corresponding HA sequence; (**2**) Structure information as well as the mutation frequency were both considered to select a set of combinational positions contributing to antigenic variance. 47 positions containing 5 spatial clusters were derived at HA protein surface through iterative screening; (**3**) Descriptors used to build the model include PSSM score describing the sequence variance at 47 positions and micro-environmental change for 5 spatial clusters of antigenicity-dominant positions; (**4**) Antigenicity variation can be calculated for any queried HA pairs.

The assumption of our model includes 1) HA protein mutate frequently at antigenicity-dominant positions to escape evolutionary pressure while surface residues interacting with antibodies may play more important role in relating to the antigenicity change than those inner ones. 2) Not only the individual residue mutation, but also the local change of the micro-environment may lead to antigenicity deviation. Thus in addition to the mutational effects at sequence level, our model take further into account of the neighboring effects of surface mutations at structure level (*See* Method *Modelling the antigenic variance*). 47 positions were identified as antigenicity-dominant positions, locating on both head and stem region on HA protein (Fig. 1). Interestingly, these positions form 5 clusters on HA surface covering 28 epitope positions defined by PDB complexes (see supplementary Table S1, Fig. S1).

Figure 1 presented the workflow of the model. Various classification and regression algorithms were evaluated (see supplementary Table S2), and linear regression was adopted to build the prediction model. For any queried HA pair (Fig. 1 step 4), a fingerprint of 67-dimention descriptors is formulated summarizing the variance at the 47 positions (Fig. 1 step 3). The first part of the 67-descriptors in step3 illustrates the evolutionary pressure at the 47 positions via PSSM, while the second part of descriptors represents the local micro-environmental change for the 5 clusters, including isoelectric point, hydrophobicity, van der waals volume and glycosylation site. The physic-chemical properties of neighboring residues within 3 Å of each antigenicity-dominant position (including the inner residues) are summarized into physical-chemical properties for each cluster.

### Model performance

As a quantitative method, our model was extensively validated through internal validation on training dataset of 3747 HA pairs during year 1968–2010 and independent data variation on 120 HA pairs during year 2011–2013. The overall quantitative correlation coefficient between the predicted and experimental antigenic distance reached 0.896 on 10-fold cross-validation and 0.827 on the 120 independent testing pairs. Then, the performance of our 47 positions was compared that of 20 positions latest proposed by Liao’s method^8^, and the correlation coefficient of Liao’s 20 positions reached 0.761 on the 120 testing set (see supplementary Fig. S2). The overall ROC of our method achieves a high AUC value of 0.894 on 120 independent testing set to classify the antigenicity similarity (see supplementary Fig. S3).

Further, our model was compared with several online programs on independent testing dataset, including Lee and Chen’s^7^, ATIVS(Liao & Lee)^8^,^10^,^15^, Lees and Shepherd’s^3^ and AntigenCO^14^ (Table 1). In addition to classification accuracy, parameters of Mathew Correlation Coefficient (MCC) and F-score were also introduced to indicate the potential bias on unbalanced data and the overall ability considering both precision and sensitivity respectively (see *Methods parameter definition*). Among the 5 peers, ATIVS (Liao) gives the best results suggesting an excellent ability in classification with accuracy of 0.800, MCC of 0.595 and F-score of 0.854. Surprisingly, our structure-based model further enhanced the performance with even higher accuracy of 0.875, MCC of 0.748, and F-score of 0.904, showing significant improvement in unbiased performance on testing samples.

**Figure 2:**
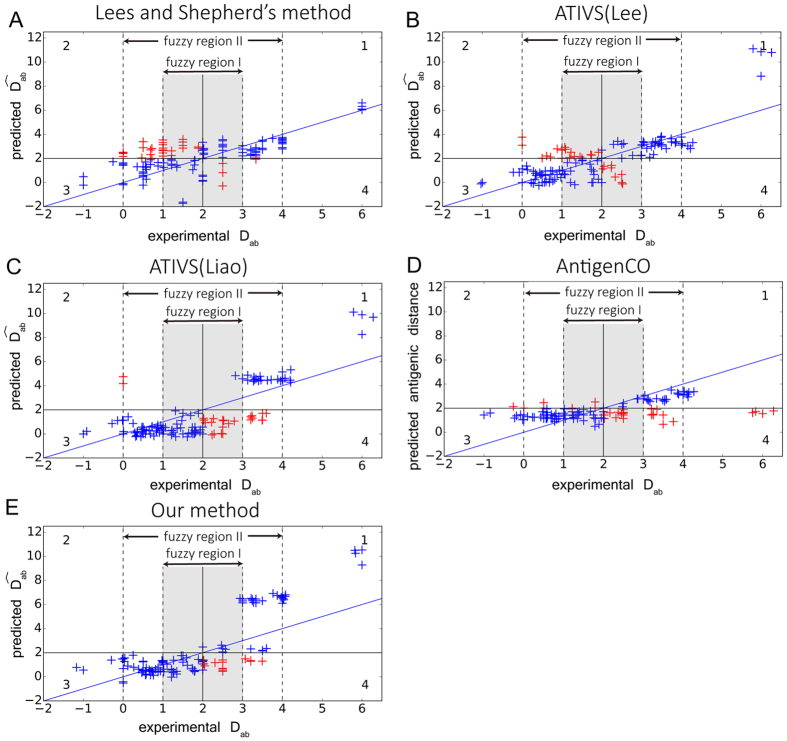
Detailed performance of representative methods on independent testing data of 120 pairs. Panel (**A**–**E**) demonstrate the correlation between predicted 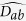 (Y-axis) and experimental *D*_*ab*_ (X-axis). In each panel, blue cross indicates those correctly classified pairs, with true negatives in quadrant 1 and true positives in quadrant 3. Red ones represent misclassified pairs, with false negatives in quadrant 2 and false positives in quadrant 4. Dotted line indicates the fuzzy regions. The fuzzy region I (*D*_*ab*_ ∈[1, 3], exclude *D*_*ab*_ = 2) was colored in gray. For better illustration, overlapped point was rearranged by slight deviation randomly without changing the overall classification. Lee and Chen’s method was a qualitative method and does not provide antigenic distance, thus was not compared in here.

To investigate the more detailed deviation between experimental and computational results, we plotted the 120 independent testing data in Fig. 2, with X-axis indicating the experimental antigenic distance in *D*_*ab*_ and Y-axis indicating the predicted antigenic distance from different models. From left to right, the increasing *D*_*ab*_ illustrates the change from similar antigenicity, across the border (log^−1^ *D*_*ab*_ = 4, *D*_*ab*_ = 2)^3^, then to antigenicity variant. We can see that the distribution of *D*_*ab*_ is highly uneven, with a condensed tendency near the border region. From our statistics, 89.167% of the testing data falls into the region of *D*_*ab*_∈[0, 4] (exclude *D*_*ab*_ = 2), and 40% of testing data falls in *D*_*ab*_ ∈[1, 3] (exclude *D*_*ab*_ = 2). We then defined a stringent fuzzy region I of *D*_*ab*_∈[1, 3] (exclude *D*_*ab*_ = 2) and clear region I as *D*_*ab*_ outside of [1, 3]. A more relaxed fuzzy region II was further defined as *D*_*ab*_∈[0, 4] (exclude *D*_*ab*_ = 2) and clear II region as *D*_*ab*_ outside [0, 4].

In Fig. 2, red cross marks the wrong predictions, and the blue marks the correct predictions. We found that prediction in both fuzzy regions seems to be more difficult than that in clear regions. The prediction accuracy of available methods range from 54.206% to 77.570% in the relaxed fuzzy II region, and 35.417% to 70.833% in the stringent fuzzy I region (see supplementary Table S3). In contrast, it’s easier to make correct predictions by all methods in the clear regions. It is reasonable because clear regions represent the highly similar or highly different antigenicity, where simply counting the number of mutations can discriminate major escaping cases in clear regions (see supplementary Fig. S4).

As the available data tends to concentrate towards the border regions where the prediction near the border is highly challenging, we focused on the detailed performance of different methods in fuzzy regions. Our method can reach the accuracy of 79.167% in fuzzy region I and 86.916% in fuzzy region II respectively. Meanwhile, the second best method is ATIVS (Liao) with accuracy of 70.833% in fuzzy I and 77.570% in fuzzy II respectively. In addition, our model provides the least root mean squared error (RMSE) among all quantitative peers in both fuzzy regions (see supplementary Table S3). Most importantly, our method reduces the false negatives to zero in quadrant 2, indicating the outstanding ability to detect antigenic escaping cases (Fig. 2E).

To evaluate the contribution of structure-based features, we tested alternative model using sequence-based features only. Table S4 indicates that incorporating structure-context features can improve the overall model performance from accuracy of 0.85 to 0.875, particularly it can enhance the prediction sensitivity of antigenic variance from 0.944 to 0.986. The robustness of our model is also tested to the deviation of modelled structures. We compared the difference of antigenic distance using 24 theoretically modelled structures instead of their crystalized HAs from PDB database. Details can be found in supplementary Table S5 and *Preprocessing of structure modeling*. The theoretical *D*_*ab*_ between modelled structures to each of 288 HAs in our dataset was calculated via our model. Similar work was done for the 24 crystalized structures. Given that 14 out of the 24 are incomplete sequences in modelling HA structures, the results showed no difference for 23 out of the 24 structures, suggesting the high robustness of our model using homology modelled structures.

### Detection of antigenic drift for vaccine strains

Because of the potential ability in detecting antigenic-escaping strains, we further applied our model to evaluate the historical data for WHO recommends vaccine strains. Every year, WHO proposes vaccine strains for southern and northern hemisphere respectively for the coming influenza season. An ideal vaccine strain would preferably provide wide protection against the majority of circulating strains during valid time^16^. Traditionally, the antigenic deviations are tested via HI assays between proposed vaccine strains and selected circulating strains. Here, we show the high-throughput way to achieve above goal via our structure-based method.

**Figure 3:**
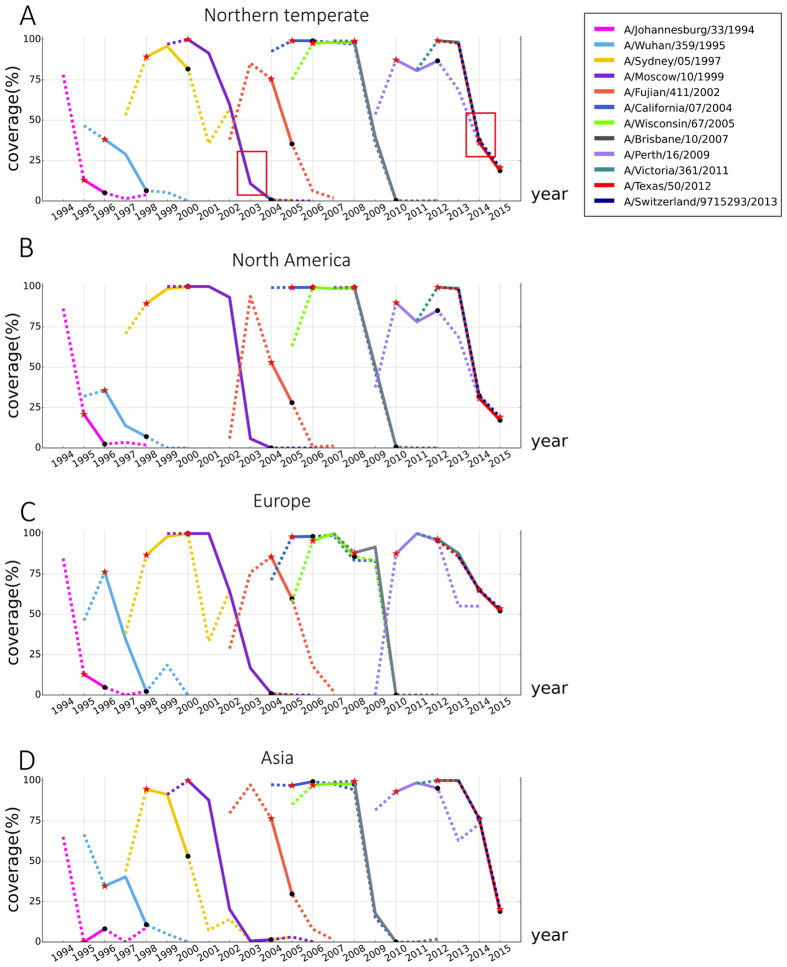
Vaccine coverage in northern temperate and three continents for the recent 20 years. In subgraph (**A**–**D**), X-axis represents years from 1994 to 2015 and Y-axis represent vaccine coverage of each year. Each line refers to the antigenicity coverage of a vaccine strain from its emerging year to two years after being replaced by updated vaccine strain. Red stars indicate the recommendation years of the vaccine strain, while black dots labels the year being updated. Red box labeled the detected vaccine failure in seasons of 2003–2004 and 2014–2015.

The theoretical antigenicity coverage was dynamically calculated for each WHO vaccine strain as the proportion of antigenic similar strains among all circulating strains in corresponding year (1994 to 2015). Figure 3 illustrates the results of northern vaccine, results of southern vaccine can be found in supplementary Fig. S5. It can be seen that most of the vaccine strains could successfully cover the antigenicity of a high portion of circulating strains (Fig. 3A), suggesting the wide efficacy during valid times. Despite of varied coverage, most vaccine strains displayed an inverted-V distribution of antigenicity coverage, with an ascending-maintaining-descending shape. Among the 12 vaccine strains, only A/Sydney/05/1997 was proposed as vaccine strain before the peak year of antigenicity coverage, and 5 vaccines were suggested after the peak year, while 6 vaccines were recommended in the year of peak coverage. Surprisingly, the antigenicity coverage of strain A/Johannesburg/33/1994 reached the highest coverage of over 78% in 1994 in northern temperate, but it was recommended as vaccine strain in 1995 when its coverage had dropped sharply. Same occurred to vaccine strain A/Fujian/411/2002, hinting a noticeable lag between the vaccine recommendation and the rapid evolution of virus antigenicity.

Interestingly, significant low coverage of vaccine strains was detected as potential vaccine failures from Fig. 3A. For instance, in the season of 2003–2004, WHO recommended A/Moscow/10/1999 as vaccine strain. However, it was reported that 87% of the isolated viruses in this season were antigenically similar to a drift variant A/Fujian/411/2002, instead of A/Moscow/10/1999^17^. In our modelled result, the extremely low theoretical coverage of A/Moscow/10/1999 was successfully detected as only 10.746%. Similarly, a very low theoretical antigenic coverage of 40.043% was also detected for vaccine strain of A/Texas/50/2012 in 2014–2015 season. The reported vaccine effectiveness in 2014–2015 were only 13% from the observational study of clinical outcomes (95% confidence interval: 2 to 23)^18^.

Similar calculation was done for peering methods of Lee and Chen’s^7^, ATIVS(Liao & Lee)^8^,^10^,^15^, Lees and Shepherd’s^3^ and AntigenCO^14^ (see supplementary Fig. S6). It can be seen some peer methods gave very low antigenicity coverage for those successful vaccine strains, even in the first year of being proposed. Examples are vaccine strain of 2000 and 2012 by Lee and Chen’s, ATIVS (Lee), Lees and Shepherd’s. Similar case is vaccine strain of 2004 and 2006 by AntigenCO. ATIVS (Liao) gave much better prediction of vaccine coverage than other peers, including the vaccine failure of 2003. But the well-known vaccine failure of 2014 was not detected by ATIVS (Liao) from Fig. S6C.

In the three main continents of northern hemisphere, most of the vaccine strains share roughly similar coverage trend (Fig. 3B–D). But at a given time, different coverage was detected in different continents for a same vaccine strain. For instance of 1995–1996, coverage of A/Wuhan/359/1995 keep increased with 75% coverage in Europe (Fig. 3C), nevertheless the coverage decreased rapidly in Asia (Fig. 3D) and the coverage remain at low level in North America as well as in northern temperate (Fig. 3A,B). Similarly, the antigenicity of A/Fujian/411/2002 covered over 75% of circulating strains in Asia when it was firstly detected in 2002, and soon rose to peak in 2003 in both Asia and North America. But it was only proposed as vaccine strain in 2004 when its antigenicity coverage peaked in Europe but dropped significantly in both Asia and North America. In 2012, Jiang’s team pointed out that the emergence time and dominance magnitude of antigenic clusters were different in different areas of the world, which agrees highly well with our findings^11^. Thus, timely proposal of regional vaccine strains according to local antigenicity evolution may help to enhance the effectiveness of vaccine measures.

## Discussion

*In-silico* evaluation of antigenicity change in influenza was proven useful to aid experimental screening, while better models have always been desired. In this paper, we constructed an improved method to calculate the antigenicity distance for H3N2 influenza incorporating structure context of HA protein. Compared with other sequence-based peer methods, our structure-based model can significantly improve the discovery rate of antigenic-escaping strains, in addition to overall prediction accuracy.

To realize that, we collected a comprehensive training dataset with 3747 pairs of HI assay values to structurally derive antigenicity-dominant positions. It is realized that different antigenic positions may be derived from different dataset and models. Despite of the difference, certain overlapping was found between our 47 positions and the previous reports. For instance, 46 positions were found to be covered by Bush’s works^4^ and 29 common positions were included by the widely used 44 positions proposed by Smith^6^. The 47 sites we identified are all on protein surface, while Smith’s sites include two buried residues (V230I and V202I). Moreover, 7 recently reported positions responsible for major antigenic change were all covered by our set^19^. Since the antigenicity-dominant positions were reported to evolve with time in HA antigen, continuous accumulation of HI data and timely updating of antigenicity-dominant positions would benefit *in silico* prediction, as is seen in this paper.

Apart from the contribution of antigenicity-dominant positions, the improved performance of our model is also contributed by full consideration of the PSSM profile and the structure context over antigenicity-dominant positions. Previous features mainly focused on sequence-based characters like the number of mutations, residual distribution, physicochemical properties of amino acids, glycosylation site and so on^3^,^7^,^11^,^12^,^13^. In this paper, the mutations are further evaluated by a profile of PSSM, which provides a more detailed description on evolution pressure at antigenicity-dominant positions at sequence level rather than the simple mutation code (binary yes or no). More importantly, neighboring residues of antigenicity-dominant positions, including surface and inner ones, are also considered to better describe the local environmental change around target position. Thus, our model is based on, but not limited to the investigated antigenicity-dominant positions.

Currently, prediction of vaccine strain has been pretty successful^11^, while early detection of vaccine failure is still challenging. Partial reason for above may be related to the unbalanced dataset. From alternative perspective, vaccine failure is mainly caused by the accumulated mutations in the HA populations which leads to the gradual escape from the host immunity. In this sense, the peak distribution of antigenic distances between new mutant strains to the previous vaccine strain is expected to drift from small deviation to larger distance, during which the antigenic distances of a fair portion of new strains would fall into nearby regions of phase change(*D*_*ab*_ = 2). Thus we defined the fuzzy regions to imply these sensitive zones, which is important for early detection of vaccine failure. Incorporating structure context can just increase the sensitivity to remove false negatives and reduce false positives during the fuzzy regions. The subsequent large-scale prediction of antigenic coverage for historical vaccines proved that our model can indeed detect the vaccine failure before experimental alert, in addition to provide regional suggestions.

Influenza’s consistent genetic drift is a well-known limitation of the vaccine and it is highly important to alter the to-be-failed vaccine in order to update vaccine strains timely. Our paper provided an improved model for antigenicity calculation incorporating structure-context of HA antigen for influenza A/H3N2 virus. Although several other factors contribute to the vaccine effectiveness, application to real-world data showed the ability of this model in sensitively identifying the vaccine failure cases, in addition to suggesting new vaccine strains. We are currently collecting HA proteins for structure modelling, and building the on-line tools. Future plan also includes building improved models for additional subtypes of influenza so that it can serve the public better.

## Methods

### Dataset

HI-assay results were firstly collected from reports of international organizations and papers. Then, antigenic distance (*D*_*ab*_) of 3867 non-redundant HA pairs were derived from 288 unique HA sequence covering 3539 strain pairs. Among which, 3747 HA pairs involving strains from 1968 to 2010 were adopted as training, while the left 120 HA pairs from 2011 to 2013 were used as the independent testing dataset.


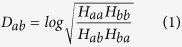


The HI titer *H*_*ab*_ is the maximum dilution of serum raised again strain *a*, which is necessary to inhibit cell agglutination caused by strain *b*. Strain *a* and strain *b* were defined as antigenic variants (negative) when the log^−1^*D*_*ab*_ was above 4, otherwise, the pair was treated as antigenic similar (positive)^3^. Further, 1848 representative HA structures were modeled by Modeller 9.11^20^ representing 18072 HA1 sequence longer than 327 amino acids from 1968 to 2015 from various international databases: The influenza virus resource at the National Center for Biotechnology Information^21^, Global Initiative on Sharing All Influenza Data (http://platform.gisaid.org/), FluKB^22^, Influenza research database^23^ and reports from National Institute for Medical Research (http://www.nimr.mrc.ac.uk/). Details can be found in supplementary Data *Collection* and *Data Preprocessing*.

### Identifying antigenicity-dominant positions

As antigenicity recognition often occurs at binding interface of antigen, we started from those surface mutations in correlation with antigenicity distance in training data. After multiple sequence alignment, each position *i* from 1 to the full alignment length 330 will be considered if:

1) The position mutated frequently (>10%) in historical monitoring.

2) Or the mutation at this particular position tends to cause immunity escape with escape rate *ER*_*i*_ ≥ 70%.


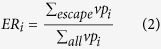


*vp*_*i*_ = 1 when a mutation occurred at position *p*_*i*_ in the HA pair, otherwise *vp*_*i*_ = 0; while 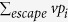 means the number of antigenically escaped pairs which all mutate at position *p*_*i*_.

After mapping those positions to template structure (PDB id: 3HMG), 100 surface positions^24^ are collected with solvent accessible surface area (SASA) over 1Å^2^, which was calculated by Naccess V2.1.1. As antigenic variation may relate to mutations at multiple positions, it can be further correlated with *D*_*ab*_ by linear regression.

3) Define position set as *P*, initial position set as *P*^(*0*)^ (*P*^(*0*)^ ⊆100 surface positions), candidate position set waiting to be screened as *P*^*c*^ (*P*^*c*^ ⊆100 surface positions), iterative position set at step *j* as *P*^(*j*)^, final key position set as *P*^(*f*)^.

4) For each HA pair with *D*_*ab*,_ a binary vector was assigned to describe the mutation at each aligned position between two HA sequences as follows:


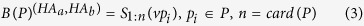


5) Then, 18 seed positions were taken as initial position set *P*^(*0*)^ by taking the consensus positions previously reported^4^,^5^,^6^ to generate initial vector B(*P*^(*0*)^) for iterative screening. 61 surface positions within 10 Å of the 18 seed positions were adopted as candidate position *P*^*c*^ for iterative screening.

6) Linear regression model: intuitively, each position is added one by one from candidates *P*^*c*^ to initial position set *P*^(*0*)^ to test their contribution to antigenicity variation, until the optimized position combination is reached as the final position set to give the best contribution to antigenicity variation. Details are described as below:

Through 10-fold cross-validation, the correlation coefficient between the predicted antigenic variance 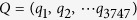 and true antigenic values of 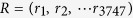 was calculated as follows. *Q* is obtained by position set and *R* is derived from HI-assay.


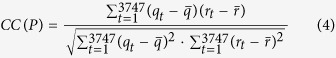


For step *j* in iterative screening, randomly add one candidate position to iterative position set:


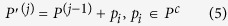


If adding this candidate position 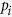 will improve the correlation coefficient 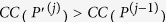 then accept this position into iterative set 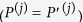 if 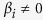, or remove those previously added positions whose 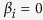 from iterative set, 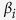 was the weight of 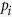.

Otherwise, reject the candidate position if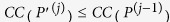, until:


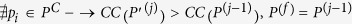


Finally, 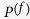 included 47 selected antigenicity-dominant positions (Fig. 1).

### Modelling the antigenic variance

#### Quantitative descriptors for antigenicity-dominant positions

Quantitative descriptors are derived covering both position-specific residue distribution and micro-environmental features for the antigenicity-dominant positions in the 3-D context. A position specific scoring matrix (PSSM) was constructed by position-specific iterated BLAST (PSI-BLAST) version 2.2.28^25^ for the 47 positions using 679 HA sequence form 1968–2010 as background (see supplementary Fig. S7).

Those antigenicity-dominant positions were clustered in 3-D space. After testing, physical-chemical AAindex^26^ of isoelectric point (ZIMJ680104), hydrophobicity (LEVM760101), and van der waals volume (FAUJ880103) were chosen as effective descriptors for further calculation, then the properties of neighboring residues within 3 Å of each key positions (including the inner residues) are summarized into physical-chemical properties for each clusters. The correlation between any two of the above index were less than 0.8. Also, mutations related to glycosylation sites were also considered for each cluster.

Finally, for the HA pair of *HA*_*a*_ and *HA*_*b*_, a 67-array quantitative descriptor for antigenicity-dominant positions (*QDAP*) was derived as below containing PSSM score and micro-environmental score (*MES*):


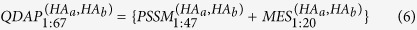



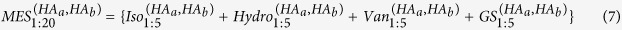



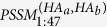
 is the absolute difference of PSSM scores for amino acids on corresponding positions. 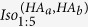,
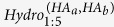
, and 
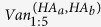
 stands for absolute difference value of the isoelectric point, hydrophobicity and van der waals volume respectively of the corresponding clusters in a HA pair. The N-linked glycosylation pattern is defined as Asn-X-Ser/Thr, where X is any amino acid apart from proline, glycan could be attached to asparagine^27^. The value of 1 or 0 would be assigned to the position according to whether it is glycosylation site or not. The cluster difference 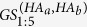 is calculated as the edit distance between two glycosylation vectors of each cluster.

#### Linear regression model

Finally, linear regression was adopted to fit the parameters of 67-dimension descriptors for antigenic variation on the 3747 pairs of training data, as follows:


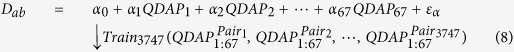


Till the optimized model is reached as below:


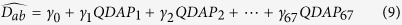


Score 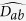 stood for the predicted antigenicity variation between two HA proteins. The experimental 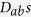 from the HI assay were used to train the model, so that the score of our model 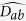 can be directly compared to experimental 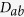. Thus the escape threshold for the predicted 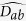 is the same as that of the 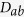 from HI assay.

### Antigenicity coverage of vaccine strain

The antigenicity coverage of each WHO recommended vaccine strain in each year was defined as below:


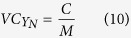


where 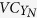 means the vaccine coverage for year *N*, *M* means the total number of emerging strains collected in year *N*, *C* means the number of antigenic similar strains of the vaccine strain in *M*.

### Parameter definition

To evaluate the performance of our model, statistical parameters were defined as follows:


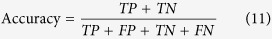



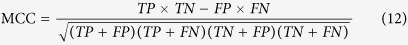



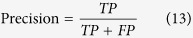



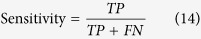



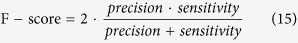


here, TP represents true positive, TN represents true negative, FP represents false positive and the FN represents false negative. Also, to evaluate our regression model, correlation coefficient was introduced as follows:


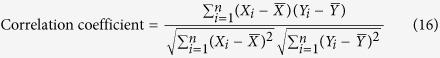


where 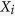 represents the predicted value, 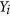 represents the actual value, 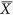 refer to average of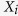, and 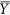 refer to the average of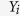.

## Additional Information

**How to cite this article**: Qiu, J. *et al.* Incorporating structure context of HA protein to improve antigenicity calculation for influenza virus A/H3N2. *Sci. Rep.* **6**, 31156; doi: 10.1038/srep31156 (2016).

## Supplementary Material

Supplementary Information

## Figures and Tables

**Table 1:** The performance of peering methods on 120 independent testing HA pairs.

Methods	Accuracy	MCC	F-score	Sensitivity	Number of positions
**Lee and Chen’s method**	0.575	0.274	0.514	0.375	131
**Lees and Shepherd’s method**	0.725	0.516	0.723	0.597	241
**AntigenCO**	0.725	0.411	0.792	0.875	38
**ATIVS(Lee)**	0.767	0.544	0.788	0.722	17
**ATIVS(Liao)**	0.800	0.595	0.854	0.972	20
**Our method**	**0.875**	**0.748**	**0.904**	**0.986**	**47**
