# Supplementary material for: Incorporating structure context of HA protein to improve antigenicity calculation for influenza virus A/H3N2
Source: Sci Rep. 2016 Aug 8;6:31156. doi: 10.1038/srep31156 (PMC4976332; doi:10.1038/srep31156)
Supplement: Supplementary Information [file srep31156-s1.pdf]

Incorporating structure context of HA protein to improve antigenicity calculation for influenza virus A/H3N2

Jingxuan Qiu[1], Tianyi Qiu[1], Yiyan Yang[1], Dingfeng Wu[1], Zhiwei Cao[1*]
[1]Department of Bioinformatics, School of Life Sciences and Technology, Tongji University, Shanghai 200092, China
[*]Correspondence should be addressed to Z.W.C. (Email: zwcao@tongji.edu.cn)

1. Data Collection

1.1 HA1 sequence collection

(1) NCBI Influenza Virus Resource[1]
(2) Global Initiative on Sharing All Influenza Data (GISAID)
http://platform.gisaid.org/
(3) Influenza Research Database (IRD) [2]
(4) FluKB[3]
(5) Reports from National Institute for Medical Research (NIMR)
http://www.nimr.mrc.ac.uk/

1.2 HI assay data collection

(1) Organization: U.S. Food and Drug Administration.
Access:
http://www.fda.gov/AdvisoryCommittees/CommitteesMeetingMaterials/BloodVaccinesandOtherBiologics/VaccinesandRelatedBiologicalProductsAdvisoryCommittee/default.htm.
Detail:
Information for the Vaccines and Related Biological Products Advisory Committee. February 22, 2010
Information for the Vaccines and Related Biological Products Advisory Committee. (Seasonal Influenza Vaccines).February 25, 2011
Information for the Vaccines and Related Biological Products Advisory Committee. (Seasonal Influenza and Zoonotic Influenza). February 28, 2012
Information for the Vaccines and Related Biological Products Advisory Committee. (Information Regarding Seasonal Influenza Viruses). February 27, 2013

(2) Organization: World Health Organization.
Access: http://www.who.int/wer/en/
Detail:
Weekly Epidemiological Record 1969 No.1 ~ No.51-52
Weekly Epidemiological Record 1970 No.1-2 ~ No.52
Weekly Epidemiological Record 1971 No.1-2 ~ No.52-53
Weekly Epidemiological Record 1972 No.1 ~ No.51-52
Weekly Epidemiological Record 1973 No.1 ~ No.52

Weekly Epidemiological Record 1974 No.1 ~ No.51-52
Weekly Epidemiological Record 1975 No.1-2 ~ No.52
Weekly Epidemiological Record 1976 No.1-2 ~ No.52
Weekly Epidemiological Record 1977 No.1 ~ No.52
Weekly Epidemiological Record 1978 No.1 ~ No.51-52
Weekly Epidemiological Record 1979 No.1 ~ No.51-52
Weekly Epidemiological Record 1980 No.1 ~ No.52
Weekly Epidemiological Record 1981 No.1 ~ No.51-52
Weekly Epidemiological Record 1982 No.1 ~ No.51-52
Weekly Epidemiological Record 1983 No.1 ~ No.51-52
Weekly Epidemiological Record 1984 No.1 ~ No.51-52
Weekly Epidemiological Record 1985 No.1 ~ No.51-52
Weekly Epidemiological Record 1986 No.1 ~ No.51-52
Weekly Epidemiological Record 1987 No.1-2 ~ No.51-52
Weekly Epidemiological Record 1988 No.1-2 ~ No.52
Weekly Epidemiological Record 1989 No.1 ~ No.51-52
Weekly Epidemiological Record 1990 No.1 ~ No.51-52
Weekly Epidemiological Record 1991 No.1-2 ~ No.51-52
Weekly Epidemiological Record 1992 No.1-2 ~ No.51-52
Weekly Epidemiological Record 1993 No.1-2 ~ No.52
Weekly Epidemiological Record 1994 No.1 ~ No.51-52
Weekly Epidemiological Record 1995 No.1 ~ No.51-52
Weekly Epidemiological Record 1996 No.1 ~ No.51-52
Weekly Epidemiological Record 1997 No.1-2 ~ No.51-52
Weekly Epidemiological Record 1998 No.1-2 ~ No.51-52
Weekly Epidemiological Record 1999 No.1~ No.51-52
Weekly Epidemiological Record 2000 No.1 ~ No.51-52
Weekly Epidemiological Record 2001 No.1 ~ No.51-52
Weekly Epidemiological Record 2002 No.1 ~ No.51-52
Weekly Epidemiological Record 2003 No.1-2 ~ No.51-52
Weekly Epidemiological Record 2004 No.1-2 ~ No.51-52
Weekly Epidemiological Record 2005 No.1 ~ No.51-52
Weekly Epidemiological Record 2006 No.1 ~ No.51-52
Weekly Epidemiological Record 2007 No.1-2 ~ No.51-52
Weekly Epidemiological Record 2008 No.1 ~ No.51-52
Weekly Epidemiological Record 2009 No.1-2 ~ No.51-52
Weekly Epidemiological Record 2010 No.1-2 ~ No.51-52
Weekly Epidemiological Record 2011 No.1-2 ~ No.51-52
Weekly Epidemiological Record 2012 No.1 ~ No.51-52
Weekly Epidemiological Record 2013 No.1 ~ No.52

(3) Organization: WHO Collaborating Centre for Reference and Research on Influenza.

Reports: Reports and newsletters.

Access: http://www.influenzacentre.org/centre_reports.htm.

Detail:
Annual report 2006
Annual report 2010
Annual report 2011
Annual report 2012

(4) Organization: Australian Government Department of Health.
Reports: National Influenza Surveillance Scheme annual reports.
Access: http://www.health.gov.au/internet/main/publishing.nsf/Content/cda-pubs-annlrpt-fluannrep.htm.
Detail:
National influenza surveillance 1994 – annual report
National influenza surveillance 1995 – annual report
National influenza surveillance 1996 – annual report
National influenza surveillance 1997 – annual report
National influenza surveillance 1998 – annual report
National influenza surveillance 1999 – annual report
National influenza surveillance 2000 – annual report
National influenza surveillance 2001 – annual report
National influenza surveillance 2002 – annual report
National influenza surveillance 2003 – annual report
National influenza surveillance 2004 – annual report
National influenza surveillance 2005 – annual report
National influenza surveillance 2006 – annual report
National influenza surveillance 2007 – annual report
National influenza surveillance 2008 – annual report

(5) Organization: Public Health Surveillance from for New Zealand.
Access: https://surv.esr.cri.nz/PDF_surveillance/Virology/FluVac/
Detail:
Recommendation for the influenza vaccine composition 2005
Recommendation for the influenza vaccine composition 2006
Recommendation for the influenza vaccine composition 2007
Recommendation for the influenza vaccine composition 2008
Recommendation for the influenza vaccine composition 2009
Recommendation for the influenza vaccine composition 2010
Recommendation for the influenza vaccine composition 2011
Recommendation for the influenza vaccine composition 2012
Recommendation for the influenza vaccine composition 2013

(6) Organization: European Centre for Disease Prevention and Control.
Access:
http://www.ecdc.europa.eu/en/PUBLICATIONS/surveillance_reports/Pages/index.aspx
Detail:
Influenza virus characterization. Summary Europe, January 2010
Influenza virus characterization. Summary Europe, February 2010

Influenza virus characterization. Summary Europe, March 2010
Influenza virus characterization. Summary Europe, April 2010
Influenza virus characterization. Summary Europe, May 2010
Influenza virus characterization. Summary Europe, June 2010
Influenza virus characterization. Summary Europe, July 2010
Influenza virus characterization. Summary Europe, August 2010
Influenza virus characterization. Summary Europe, September 2010
Influenza virus characterization. Summary Europe, December 2010
Influenza virus characterization. Summary Europe, February 2011
Influenza virus characterization. Summary Europe, March 2011
Influenza virus characterization. Summary Europe, April 2011
Influenza virus characterization. Summary Europe, May-June 2011
Influenza virus characterization. Summary Europe, July 2011
Influenza virus characterization. Summary Europe, August and September 2011
Influenza virus characterization. Summary Europe, December 2011
Influenza virus characterization. Summary Europe, February 2012
Influenza virus characterization. Summary Europe, March 2012
Influenza virus characterization. Summary Europe, June 2012
Influenza virus characterization. Summary Europe, July 2012
Influenza virus characterization. Summary Europe, September 2012
Influenza virus characterization. Summary Europe, October 2012
Influenza virus characterization. Summary Europe, November 2012
Influenza virus characterization. Summary Europe, December 2012
Influenza virus characterization. Summary Europe, February 2013
Influenza virus characterization. Summary Europe, March 2013
Influenza virus characterization. Summary Europe, April 2013
Influenza virus characterization. Summary Europe, May 2013
Influenza virus characterization. Summary Europe, June 2013
Influenza virus characterization. Summary Europe, July 2013
Influenza virus characterization. Summary Europe, September 2013

(7) Organization: National Institute for Medical Research.

Access:
http://www.nimr.mrc.ac.uk/who-influenza-centre/annual-and-interim-reports/

Detail:
Annual report 2002
Annual report 2003
Annual report 2004
Interim Report February 2005
Interim Report September 2005
Interim Report March 2006
Interim Report September 2006
Interim Report March 2007
Interim Report September 2007
Interim Report March 2008

Interim Report September 2008
Interim Report February 2009
Interim Report September 2009
Interim Report February 2010
Interim Report September 2010
Interim Report February 2011
Interim Report September 2011
Interim Report February 2012
Interim Report September 2012
Interim Report February 2013
Interim Report September 2013

(8) Published papers

Baek, Y. H. et al. Molecular characterization and phylogenetic analysis of H3N2 human influenza A viruses in Cheongju, South Korea. Journal of microbiology 47, 91-100, doi:10.1007/s12275-008-0207-y (2009).

Pechirra, P., Goncalves, P., Arraiolos, A., Coelho, A. & Rebelo-de-Andrade, H. Characterization of influenza A/Fujian/411/2002(H3N2)-like viruses isolated in Portugal between 2003 and 2005. Journal of medical virology 80, 1624-1630, doi:10.1002/jmv.21258 (2008).

de Jong, J. C. et al. Antigenic and genetic evolution of swine influenza A (H3N2) viruses in Europe. Journal of virology 81, 4315-4322, doi:10.1128/JVI.02458-06 (2007).

Iorio, A. M. et al. An influenza A/H3 outbreak during the 2004/2005 winter in elderly vaccinated people living in a nursing home. Vaccine 24, 6615-6619, doi:10.1016/j.vaccine.2006.05.037 (2006).

Daum, L. T. et al. Influenza A (H3N2) outbreak, Nepal. Emerging infectious diseases 11, 1186-1191, doi:10.3201/eid1108.050302 (2005).

Campitelli, L. et al. H3N2 influenza viruses from domestic chickens in Italy: an increasing role for chickens in the ecology of influenza? The Journal of general virology 83, 413-420, doi:10.1099/0022-1317-83-2-413 (2002).

Hay, A. J., Gregory, V., Douglas, A. R. & Lin, Y. P. The evolution of human influenza viruses. Philosophical transactions of the Royal Society of London. Series B, Biological sciences 356, 1861-1870, doi:10.1098/rstb.2001.0999 (2001).

Coiras, M. T. et al. Rapid molecular analysis of the haemagglutinin gene of human influenza A H3N2 viruses isolated in spain from 1996 to 2000. Archives of virology 146, 2133-2147 (2001).

Ellis, J. S., Chakraverty, P. & Clewley, J. P. Genetic and antigenic variation in the haemagglutinin of recently circulating human influenza A (H3N2) viruses in the United Kingdom. Archives of virology 140, 1889-1904 (1995).

Castrucci, M. R. et al. Antigenic and sequence analysis of H3 influenza virus haemagglutinins from pigs in Italy. The Journal of general virology 75 (Pt 2), 371-379, doi:10.1099/0022-1317-75-2-371 (1994).

Nakajima, S., Takeuchi, Y. & Nakajima, K. Location on the evolutionary tree of influenza H3 haemagglutinin genes of Japanese strains isolated during 1985-6

season. Epidemiology and infection 100, 301-310 (1988).
Both, G. W., Sleigh, M. J., Cox, N. J. & Kendal, A. P. Antigenic drift in influenza virus H3 hemagglutinin from 1968 to 1980: multiple evolutionary pathways and sequential amino acid changes at key antigenic sites. Journal of virology 48, 52-60 (1983).

2. Data Preprocessing

2.1 Preprocessing of HA1 sequence

In order to align all the sequences in our dataset into a unified length, the multiple alignment was done with the HA1 sequence of A/Aichi/2/1968 (H3N2) selected as a template. The alignment part of the sequence ranging from the start position of template sequence to the last position was fetched. Those with the alignment part less than 327 amino acid were excluded and the sequences belonged to the same strain with the exact same alignment part were only kept one in our dataset. The amino acids were numbered according to A/Aichi/2/1968(H3N2) HA1 sequence. Totally, 18072 HA1 sequence longer than 327 amino acids from 1968 to 2015 were collected.

2.2 Preprocessing of antigenic distance parameter

The antigenic distance between two strains a and b was defined by Lapedes and Farber in 2001 as following equation[4]:

$$D_{ab} = log\sqrt{\frac{H_{aa}H_{bb}}{H_{ab}H_{ba}}}$$

The HI titer H_{ab} is the maximum dilution of serum raised against strain a, which is necessary to inhibit cell agglutination caused by strain b. D_{ab} was calculated only if four HI values were available(H_{aa},H_{bb},H_{ab},H_{ba}). For the HI value with ">" or "<", the double or half of the value were used. For example, the HI value of ">2560" were treated as 2560*2=5120, the HI value of "<20" were treated as 20/2=10. Due to the different experimental conditions, for the same strain pair, the HI measures collected from different reports existed difference. To avoid this affection, for the HI values of same strain pair derived from different reports, the outlines defined as those with$|D_{ab} - \overline{D_{ab}}|$ ranked within top 10% in descending order were abandoned. The final experimental distance between two strains was defined as the average of those remained D_{ab}. Two viruses were defined as antigenic variants when the $log^{-1}D_{ab}$ was above 4, otherwise, the pair was treated as antigenic similar[5].

Finally, the intersection of the HI assays and the sequence set were generated as our dataset. This set contains 3867 pairs involving 288 HA proteins with 2286 antigenic variants and 1581 antigenic similar. Data ranging from 2011 to 2013 were selected as the independent dataset to evaluate the performance of this model.

2.3 Preprocessing of structure modeling

To describe the spatial features of HA protein, the three-dimensional structures were generated by Modeller 9.11[6]. For all the target sequences, the top 5 templates which

shared the high sequence identity from Protein Data Bank[7] were the same: 2VIU_A (pdb_id:2VIU, chain: A), 1HA0_A, 2VIR_C, 1MQM_A and 3EYK_A. Five models were generated based on each template, the coordination of each residue was calculated as the average of the coordination of atoms included. Then the general structure of each protein was produced by averaging the previous five models.

18072 collected HA1 sequences were clustered according to sequence identity of 99%. 1848 cluster representatives were randomly selected with one from each cluster and structures were then modeled.

3 Figures

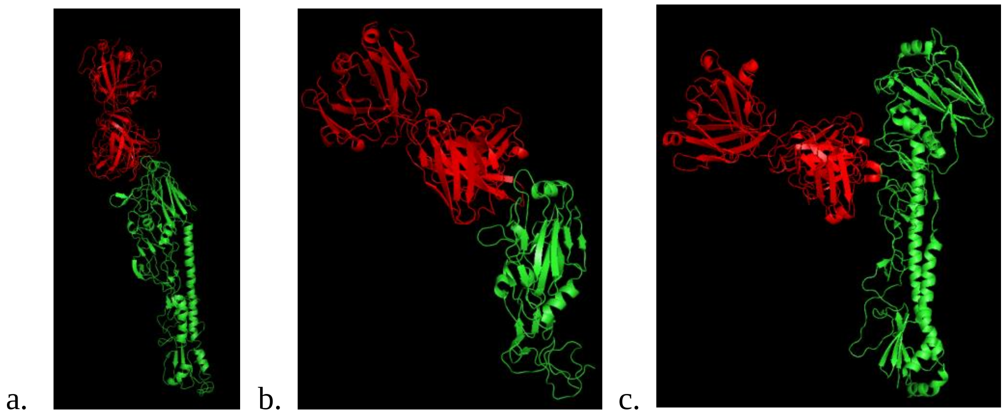

Figure. S1. Examples of PDB structure with A/H3N2 hemagglutinin bound to antibody. a: 1KEN.b: 2VIR.c: 3WHE. The hemagglutinin was marked in green with the antibody labeled red. Panel a and b demonstrated the antibodies bound to the head of HA, and panel c showed the antibody bound to the stem of HA.

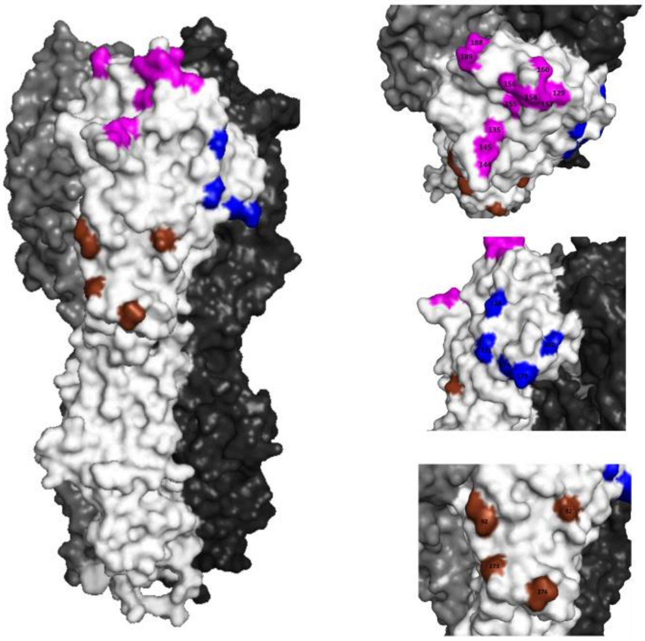

Figure. S2. The spatial location of Liao’s sites. Three clusters generated by using the

coordination distance were labeled by different colors. The detailed locations were demonstrated on the enlarged scale.

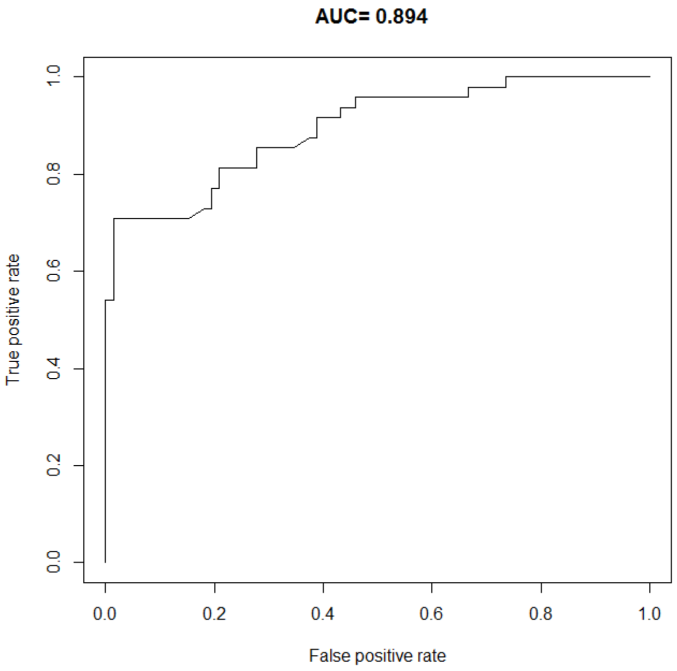

Figure.S3. Receiver operating characteristic curve (ROC) of our model on 120 independent test dataset.

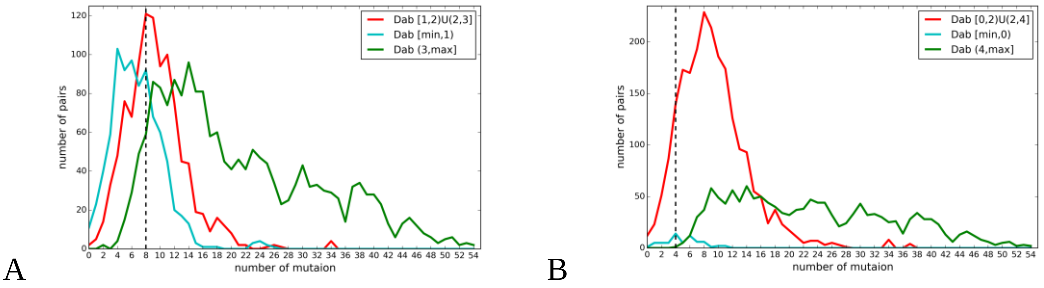

Figure. S4. Mutation number distribution of HA pairs on training dataset with D_{ab} on different intervals. X axis refers to the number of mutation and Y axis refers to the number of pairs with corresponding mutation number. Different color represented the HA pairs with D_{ab} on different intervals. Dashed line labeled the threshold of mutation number for classification. In panel A, 85.075% pairs could be correctly classified in clear region I ($D_{ab}\in$[min,1)U(3,max]) by using 8 mutations as escaping threshold. Whereas in the corresponding fuzzy region I ($D_{ab}\in$[1,2)U(2,3]), only 61.412% pairs can be rightly picked under the same cutoff. In panel B, 97.614% pairs can be correctly classified in clear region II ($D_{ab}\in$[min,0)U(4,max]) with 4 mutations as threshold. However, under this cutoff, only 48.316% pairs could be rightly picked in the fuzzy region II($D_{ab}\in$[0,2)U(2,4]).

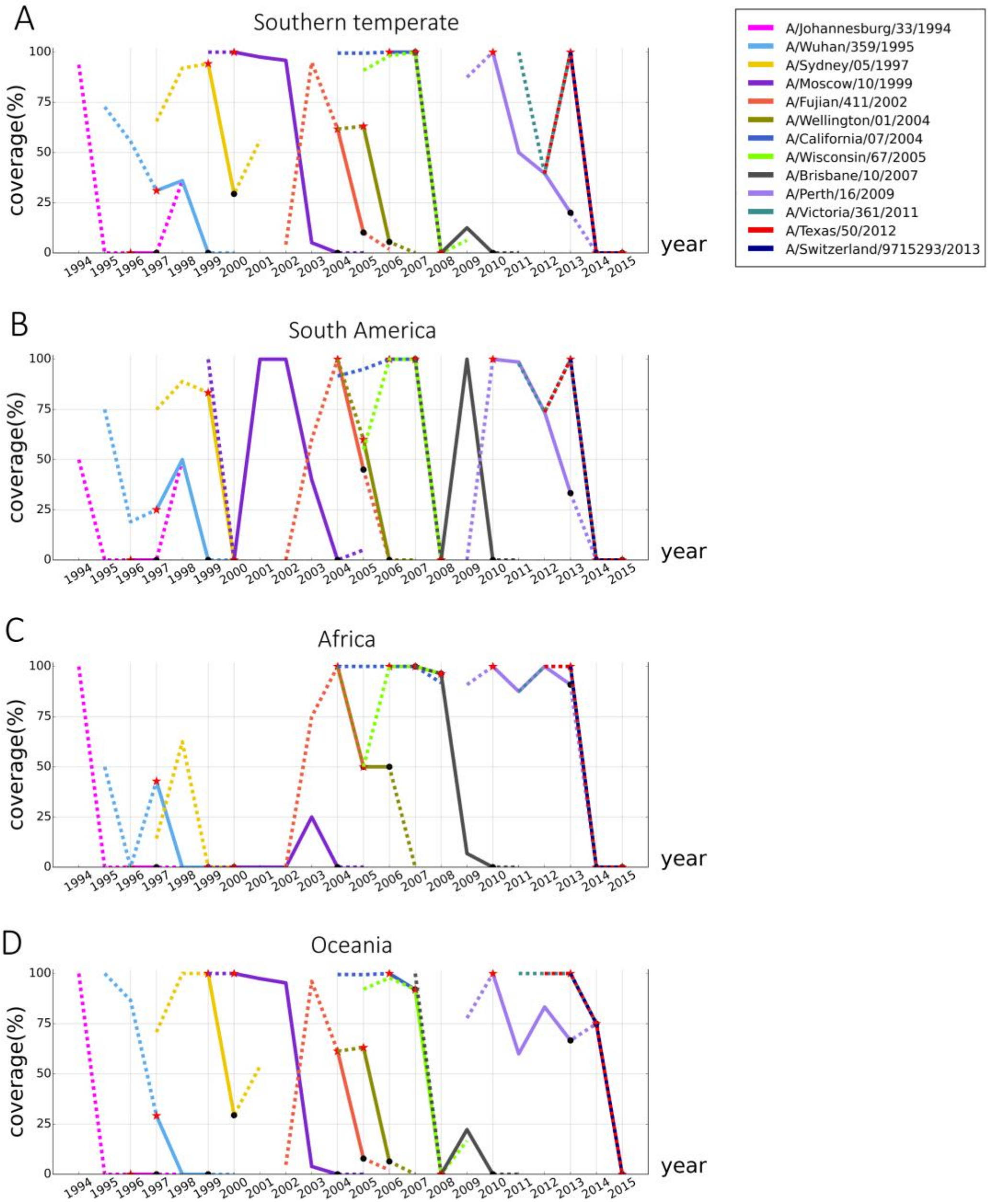

Figure. S5. Vaccine coverage in southern temperate and three continents for the recent 20 years. In subgraph A-D, X-axis represents years from 1994 to 2015 and Y-axis represents vaccine coverage of each year. Each line refers to the antigenicity coverage of a vaccine strain from its emerging year to two years after it has been replaced by the next vaccine strain. Red stars indicates the recommendation year of the vaccine strain, while black dots labels the year of its replacement.

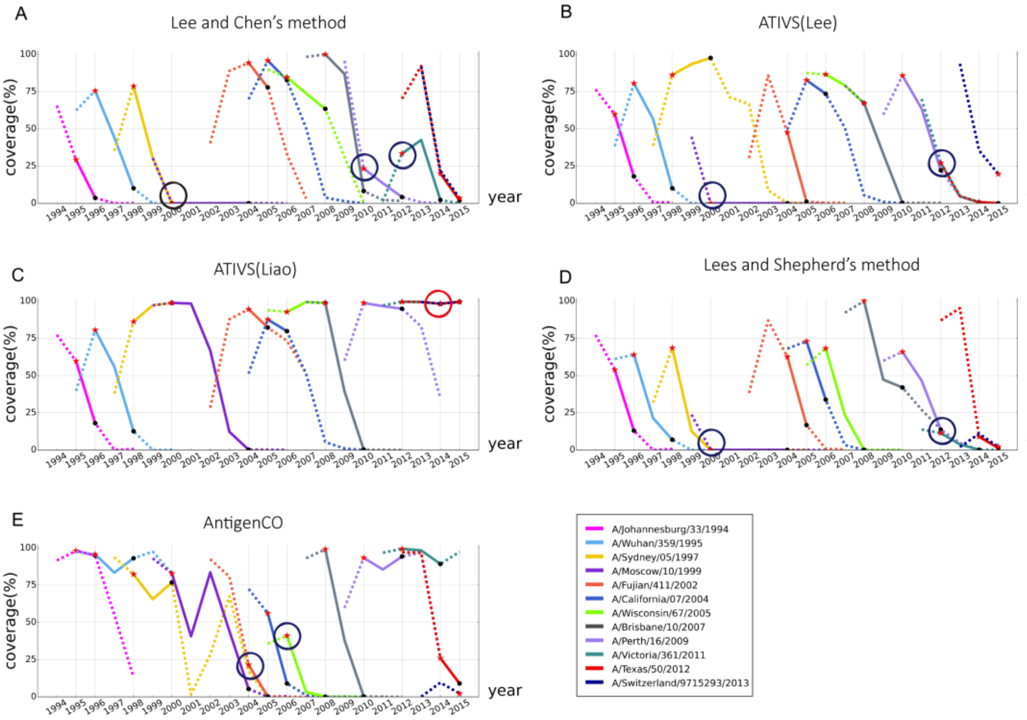

Figure S6. Vaccine coverage of latest 20 years in northern temperate calculated by peer methods. In subgraph A-E, X-axis represents years from 1994 to 2015 and Y-axis represents vaccine coverage of each year. Each line refers to the antigenicity coverage of a vaccine strain from its emerging year to two years after it has been replaced by the next vaccine strain. Red stars indicates the recommendation year of the vaccine strain, while black dots labels the year of its replacement. Red circle labeled the undetected vaccine failure. Black circle labeled the undetected vaccine effectiveness.

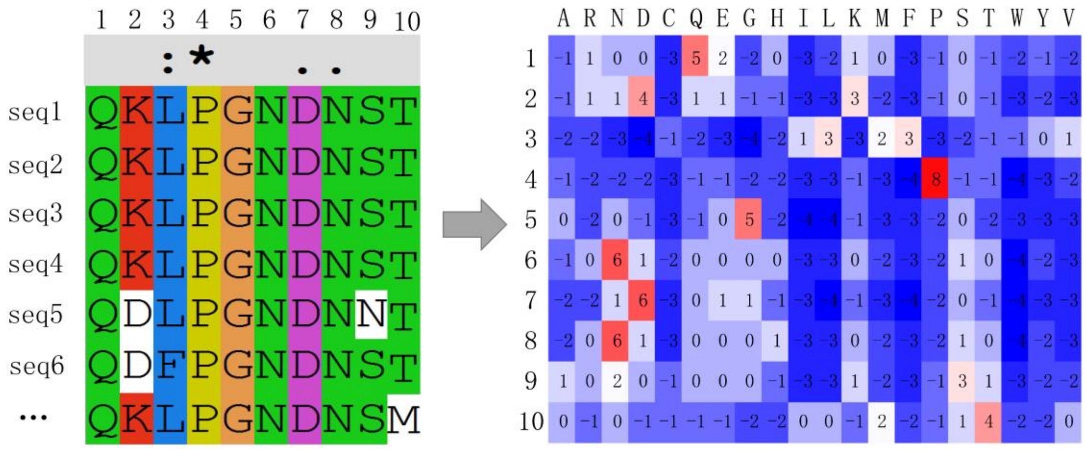

Figure. S7. Position specific scoring matrix (PSSM) construction based on multiple sequence alignment. PSSM was generated during the Position-Specific Iterated BLAST (PSI-BLAST) process based on the multiple sequence alignment of HA sequences. The matrix contained the scores of twenty amino acids to reflect the amino acid distribution on each position. For a pair of HA, the score on a position was assigned as the absolute

difference between the scores of amino acids on the position. In case of the comparison between the gap or X and one amino acid, the maximum or average among all absolute difference between this amino acid and all the others on the position was appointed as the score. The score of amino acid Asx (B) was treated as the average of those of Asn (N) and Asp (D).

4. Tables

Table S1. List of screened 47 antigenicity-dominant positions. This table listed the selected antigenicity-dominant positions and the clusters they belonged. Definition of mutation rate and escape ratio see Method *Identifying antigenicity-dominant positions* 1) and 2).The last column listed the PDB structure (PDB id and HA chain) in which this position was epitope position. In HA protein, epitope positions were defined as those residues for which the least atom distance with antibody was less than 5 Å. Position number was label on A/Aichi/2/1968 HA1 sequence (PDB id: 3HMG_A).

Position	Cluster	Mutation rate	Escape ratio	Epitope in PDB
50	E	0.286	0.767	1EO8_A,3WHE_A,1QFU_A
57	E	0.059	0.782	
121	D	0.096	0.989	
122	D	0.188	0.677	
124	D	0.173	0.931	
129	B	0.015	0.964	2VIR_C,2VIS_C,2VIT_C
131	B	0.165	0.931	4O58_A,2VIR_C,4FP8_A,4O5I_A,2VIS_C,4FQR_A,2VIT_C,4GMS_A
132	B	0.010	0.872	4O58_A,2VIR_C,4FP8_A,4O5I_A,2VIS_C,2VIT_C
133	B	0.207	0.997	4O58_A,2VIR_C,4FP8_A,4O5I_A,2VIS_C,4FQR_A,2VIT_C
135	C	0.157	0.956	4O58_A,2VIR_C,1KEN_A,4FP8_A,4O5I_A,2VIS_C,4FQR_A,2VIT_C,4GMS_A
137	C	0.214	0.891	4O58_A,2VIR_C,1KEN_A,4FP8_A,4O5I_A,2VIS_C,4FQR_A,2VIT_C,4GMS_A
140	C	0.123	0.610	
142	C	0.122	0.643	1EO8_A,1QFU_A
143	C	0.117	0.986	1QFU_A
144	C	0.371	0.797	
145	C	0.330	0.854	4O58_A,2VIR_C,4FP8_A,4O5I_A,2VIS_C,4FQR_A,2VIT_C,4GMS_A
146	C	0.111	0.990	4O5I_A
152	B	0.013	0.800	
155	B	0.257	0.969	4O58_A,2VIR_C,4FP8_A,4O5I_A,2VIS_C,4FQR_A,2VIT_C,4GMS_A
156	B	0.370	0.817	4O58_A,2VIR_C,1KEN_A,4FP8_A,4O5I_A,2VIS_C,4FQR_A,2VIT_C,4GMS_A
157	B	0.144	0.794	4O58_A,2VIR_C,4FP8_A,2VIS_C,4FQR_A,2VIT_C,4GMS_A
158	B	0.307	0.952	4O58_A,2VIR_C,1KEN_A,4O5I_A,2VIS_C,2VIT_C,4GMS_A
159	B	0.235	0.856	4O58_A,2VIR_C,1KEN_A,4O5I_A,2VIS_C,4FQR_A,2VIT_C,4GMS_A
160	B	0.165	0.832	2VIR_C,2VIS_C,2VIT_C,4GMS_A
164	B	0.074	0.982	
172	D	0.192	0.847	
173	D	0.339	0.768	

188	A	0.139	0.703	4FQR_A
189	A	0.431	0.952	4O58_A,1KEN_A,4FP8_A,4FQR_A,4GMS_A
190	A	0.208	0.763	2VIR_C,1KEN_A,4FP8_A,2VIS_C,4FQR_A,2VIT_C,4GMS_A
193	A	0.330	0.817	4O58_A,2VIR_C,1KEN_A,4FP8_A,4O5I_A,2VIS_C,4FQR_A,2VIT_C,4GMS_A
196	B	0.145	0.689	4GMS_A
197	B	0.155	0.997	
207	D	0.067	0.960	
208	D	0.049	0.813	
216	A	0.054	0.791	
217	A	0.080	0.993	
219	A	0.128	0.626	1KEN_A
225	C	0.160	0.671	1KEN_A,4FQR_A,4GMS_A
226	C	0.337	0.768	4O58_A,2VIR_C,1KEN_A,4FP8_A,4O5I_A,2VIS_C,4FQR_A,2VIT_C,4GMS_A
240	D	0.010	0.947	
244	B	0.101	0.952	
260	D	0.152	0.804	
275	E	0.096	0.838	1QFU_A
276	E	0.129	0.979	1QFU_A
278	E	0.169	0.945	4KVN_A,3ZTJ_A
279	E	0.010	0.889	

Table S2. Performance of several classification and regression methods using quantitative descriptors on predicting antigenic distance.10-fold cross validation was tested on the training dataset from 1968 to 2010 and the independent test was conducted on the test dataset from 2011 to 2013.

Classification Approach	10-fold cross validation		Independent test set		Regression Approach	10-fold cross validation	Independent test set
	AUC[a]	Accuracy(%)	AUC	Accuracy(%)		CC[b]	CC
**BayesNet**	0.966	91.246	0.837	79.167	**Least Med Sq**	0.886	0.814
**LibSVM**	0.956	95.330	0.792	82.500	**Linear regression**	0.896	0.827
**Logistic**	0.974	92.928	0.885	82.500	**Pace regression**	0.896	0.828
**Simple logistic**	0.972	92.634	0.894	85.833	**PLS Classifier**	0.891	0.817
**Rotation Forest**	0.988	96.637	0.845	79.167	**SMO regression**	0.892	0.828
**Random Forest**	0.985	96.024	0.862	80.000	**Grid Search**	0.885	0.817

[a]AUC: area under the curve

[b]CC: correlation coefficient

Table S3. Accuracy and root mean squared error (RMSE) for mis-classified pairs of different methods in different regions of D_{ab}.

		Lee and Chen's method	Lees and Shepherd's method	ATIVS (Lee07)	ATIVS (Liao)	AntigenCO	Our method
**accuracy (%)**	fuzzy region I	35.417	60.417	58.333	70.833	68.750	79.167
	fuzzy region II	54.206	71.028	77.570	77.570	74.766	86.916
**RMSE**	fuzzy region I	*	1.741	1.431	1.749	*	1.423
	fuzzy region II	*	1.901	1.701	2.171	*	1.570

* Lee and Chen's method was a classification model without predicted $\widehat{D_{ab}}$ and the predicted antigenic distance of AntigenCO is different from D_{ab} we used here, thus the RMSE was not calculated for these two methods.

Table S4. Performance of sequence-based feature (PSSM) and structure-based feature (micro-environment change) on independent test dataset.

	Accuracy	MCC	F-score	Sensitivity
**Sequence-based features**	0.850	0.688	0.883	0.944
**Sequence-based features and Structure-based features**	0.875	0.748	0.904	0.986

Table S5. The PDB ID and chain name of 24 non-redundant crystalized HA structures used to compare the robustness of modelled structure.

PDB_ID	PDB_ID	PDB_ID	PDB_ID
1HGD_A	3VUN_A	2VIT_C	4WE4_A
1EO8_A	4O5N_A	4WE6_A	3WHE_A
2YP4_A	4FQR_A	4KVN_A	4O58_A
4FP8_A	4WE7_A*	4GMS_A	4HMG_A
4WE8_A	2VIS_C	4WE5_A	2HMG_A
2YP7_A	4UBD_A	3ZTJ_A	4ZCJ_A

* Among 24 structures, only one structure (PDB: 4WE7_A) was found that there is a difference between the antigenic distance calculated by using PDB structure and modeled structure. The averaged absolute error of all tested $D_{ab}s$ was 0.16, and the median $\Delta_{D_{ab}}$ was 0.181.

Reference

1 Bao, Y. *et al.* The influenza virus resource at the National Center for Biotechnology Information. *Journal of virology* **82**, 596-601, doi:10.1128/JVI.02005-07 (2008).

2 Squires, R. B. *et al.* Influenza research database: an integrated bioinformatics resource for influenza research and surveillance. *Influenza and other respiratory viruses* **6**, 404-416, doi:10.1111/j.1750-2659.2011.00331.x (2012).

3 Simon, C. *et al.* FluKB: A Knowledge-Based System for Influenza Vaccine Target Discovery and Analysis of the Immunological Properties of Influenza Viruses. *Journal of immunology research* **2015**, 380975, doi:10.1155/2015/380975 (2015).

4 Lapedes, A. & Farber, R. The geometry of shape space: application to influenza. *Journal of theoretical biology* **212**, 57-69, doi:10.1006/jtbi.2001.2347 (2001).

5 Lees, W. D., Moss, D. S. & Shepherd, A. J. A computational analysis of the antigenic properties of haemagglutinin in influenza A H3N2. *Bioinformatics* **26**, 1403-1408, doi:10.1093/bioinformatics/btq160 (2010).

6 Eswar, N. *et al.* Comparative protein structure modeling using Modeller. *Current protocols in bioinformatics / editoral board, Andreas D. Baxevanis ... [et al.]* **Chapter 5**, Unit 5 6, doi:10.1002/0471250953.bi0506s15 (2006).

7 Berman, H. M. *et al.* The Protein Data Bank. *Nucleic acids research* **28**, 235-242 (2000).
